# Multiparametric MRI for Prostate Cancer Detection: New Insights into the Combined Use of a Radiomic Approach with Advanced Acquisition Protocol

**DOI:** 10.3390/cancers12020390

**Published:** 2020-02-07

**Authors:** Serena Monti, Valentina Brancato, Giuseppe Di Costanzo, Luca Basso, Marta Puglia, Alfonso Ragozzino, Marco Salvatore, Carlo Cavaliere

**Affiliations:** 1 IRCCS SDN, 80143 Naples, Italy; smonti@sdn-napoli.it (S.M.); lbasso@sdn-napoli.it (L.B.); direzionescientifica@sdn-napoli.it (M.S.); ccavaliere@sdn-napoli.it (C.C.); 2 Ospedale S. Maria delle Grazie, 80078 Pozzuoli, Italy; giupe7700@yahoo.it (G.D.C.); martapuglia@alice.it (M.P.); alfonsoragozzino@gmail.com (A.R.)

**Keywords:** prostate cancer, PI-RADS, radiomics, magnetic resonance imaging, diffusion kurtosis imaging, dynamic contrast-enhanced magnetic resonance imaging

## Abstract

Prostate cancer (PCa) is a disease affecting an increasing number of men worldwide. Several efforts have been made to identify imaging biomarkers to non-invasively detect and characterize PCa, with substantial improvements thanks to multiparametric Magnetic Resonance Imaging (mpMRI). In recent years, diffusion kurtosis imaging (DKI) was proposed to be directly related to tissue physiological and pathological characteristic, while the radiomic approach was proven to be a key method to study cancer imaging phenotypes. Our aim was to compare a standard radiomic model for PCa detection, built using T2-weighted (T2W) and Apparent Diffusion Coefficient (ADC), with an advanced one, including DKI and quantitative Dynamic Contrast Enhanced (DCE), while also evaluating differences in prediction performance when using 2D or 3D lesion segmentation. The obtained results in terms of diagnostic accuracy were high for all of the performed comparisons, reaching values up to 0.99 for the area under a receiver operating characteristic curve (AUC), and 0.98 for both sensitivity and specificity. In comparison, the radiomic model based on standard features led to prediction performances higher than those of the advanced model, while greater accuracy was achieved by the model extracted from 3D segmentation. These results provide new insights into active topics of discussion, such as choosing the most convenient acquisition protocol and the most appropriate postprocessing pipeline to accurately detect and characterize PCa.

## 1. Introduction

Prostate cancer (PCa) is the second most common malignant neoplasm among men [1]. The early detection and grading of PCa are crucial for patient management and long-term survival evaluation.

The prostate cancer screening paradigm commonly consists of a serum prostate-specific antigen (PSA) test, a digital rectal examination, a transrectal ultrasound, and prostatic biopsies, but each of these methods has its disadvantages, spanning from low accuracy, i.e., when PSA is used alone, to invasiveness, i.e., in transrectal examinations or biopsies.

Recently, the use of a multiparametric Magnetic Resonance Imaging (mpMRI) approach, combining anatomic T1 or T2-weighted (T2W) images with functional MRI methods as Diffusion Weighted Imaging (DWI) and Dynamic Contrast Enhanced (DCE) imaging, provided substantial improvements in non-invasive prostate cancer detection and characterization [2,3,4]. In fact, the imaging approach, besides its non-invasiveness, can give “in vivo” information on the entire tumor volume, thereby reducing inaccuracies due to sampling errors in histopathological analyses.

The Prostate Imaging-Reporting and Data System (PI-RADS) was developed in 2013, and then updated in 2015 (PI-RADS v2) [5], in order to standardize the use of mpMRI in PCa imaging. This technology provides a scale indicating how likely an mpMRI finding from T2W, DWI, and DCE is related to a clinically significant cancer. The PI-RADS score ranges from one, which indicates a very low probability of malignancy, to five, which indicates a very high probability that a lesion is malignant. Since its introduction, the PI-RADS classification has played a very important role in PCa diagnosis [6], proving to be a useful tool for the detection of prostatic lesions and their characterization in terms of aggressiveness. However, due to its definition, PI-RADS scoring can be affected by subjectivity and inter-/intra-operator variability [7], which are factors that may compromise PCa assessment. Moreover, it is not unusual to find benign and malignant lesions with similar imaging findings, making it challenging to detect the nature of prostatic lesions [8]. It should also be considered that lesions classified as having a PI-RADS score of three are usually lesions termed as “intermediate” or “equivocal on the presence of clinically significant cancer” [9]. The abovementioned limitations of PI-RADS, together with an ever-growing volume of medical images for each patient and the development of increasingly powerful image acquisition and processing techniques, have led to an increasing interest in new quantitative approaches to analyze mpMRI images [10]. 

In particular, non-Gaussian diffusion models were proposed to better describe diffusion signal behaviors, which are directly related to tissue physiological and pathological characteristics, and to overcome the possible limitation of standard DWI. One of the most used models in the field of prostate cancer is diffusion kurtosis imaging (DKI), although this technique requires more advanced MRI sequences, longer acquisition time, and specific postprocessing tools compared with standard DWI. DKI parameters, namely, the diffusion coefficient D and the deviation from normal distribution coefficient K, proved to be very useful for PCa detection and characterization [11,12,13,14].

On the other hand, a radiomic approach proved to be a key method to study cancer imaging phenotypes, reflecting underlying clinical and pathological information, as well as gene expression patterns [15,16,17,18]. Radiomics, in fact, refers to the extraction of a large number of quantitative features from medical images [19], thereby revealing heterogeneous tumor metabolism and anatomy [20,21]. This high-throughput extraction is preparatory to a process of data mining [17] for studies of associations with or predictions of different clinical outcomes [22], thereby giving important prognostic information about disease. The potential of radiomics to extensively characterize intratumoral heterogeneity has shown promise regarding the prediction of treatment responses and outcomes, differentiating benign and malignant tumors, and assessing genetic relationships in many cancer types [23,24].

Several radiomic studies were performed to discriminate PCa from noncancerous tissues, to differentiate between cancers with different Gleason scores, and also to compare radiomic diagnostic capabilities with those of PI-RADS scores [8,25,26,27,28]. All of these studies computed radiomic features from standard mpMRI acquisition, including T2W, Apparent Diffusion Coefficient (ADC) computed from a classical Gaussian diffusion model, and eventually DCE images. Feature extraction starting from 2D regions of interest (ROIs) was performed in some of these studies, while 3D volumes of interest (VOI) was utilized in others. Only few works, to the best of our knowledge, applied radiomic approach to DKI imaging; Wang et al. [29] investigated whether radiomic features extracted from T2W, ADC, D, K, and the quantitative perfusion parameter K^trans^ could help to improve the diagnostic performance of structured PI-RADS v2 in clinically relevant PCa. In the study by Hectors et al. [30], radiomics features extracted from T2W, ADC, and DKI maps were correlated with Gleason score, gene expression signatures and cancer-related gene expression levels. Toivonen et al. [31] developed and evaluated a classification system for Gleason score predictions using radiomics features from T2W, DWI, and DKI. However, none of these fully investigated the added value of combining the complex radiomic processing approach with the advanced DKI mathematical model for PCa detection.

The aim of our study was to fully investigate this issue. We obtained mpMRI radiomic signatures to discriminate between 4–5 PI-RADS PCa and healthy tissue (HT) in a standard model, computed using T2W and ADC alone, and using more advanced technology, which included using DKI and quantitative DCE pharmacokinetic parameters [32]. Besides comparing the accuracies obtained by these two radiomic signatures, we also evaluated the differences found in prediction performances when using 2D ROIs or 3D VOIs.

## 2. Results

First and second order radiomic features (described in detail in the Materials and Methods section) were extracted from T2W, ADC, DKI, and quantitative DCE pharmacokinetic parameters (volume transfer constant from the plasma compartment to the extravascular extracellular space, K^trans^; rate constant for transfer between the extravascular extracellular space and the blood compartment, K_ep_; volume of extravascular extracellular space per unit volume of tissue, v_e_; the initial area under the enhancement curve, iAUC).

The initial sets of features were composed of 120 features for two of the three analyzed classification tasks, i.e., PI-RADS 4–5 vs. HT on 3D VOI in the advanced model (adv3D), which was computed using first order features from T2W, ADC, D, K, and quantitative DCE pharmacokinetic parameters, and PI-RADS 4–5 vs. HT on 2D ROI in the advanced model (adv2D). The third feature set was composed of 44 features for the classification task PI-RADS 4–5 vs. HT on 3D VOI in the standard model (std3D), which was computed using first and second order features from T2W and ADC. 

Considering that, for a single patient, more than one segmentation can be obtained, i.e., both lesion and HT can be obtained from the same prostate and more lesions or more healthy regions in the same subject, a total of 118 3D VOIs were segmented (69 PCa and 49 HT); correspondingly, 118 2D ROIs were extracted.

For each classification task, a reduced feature set was computed according to a stepwise forward feature selection scheme. Each feature set, as reported in Table 1, was composed of the 25 top-ranked features in the gain equation.

**Figure 1:**
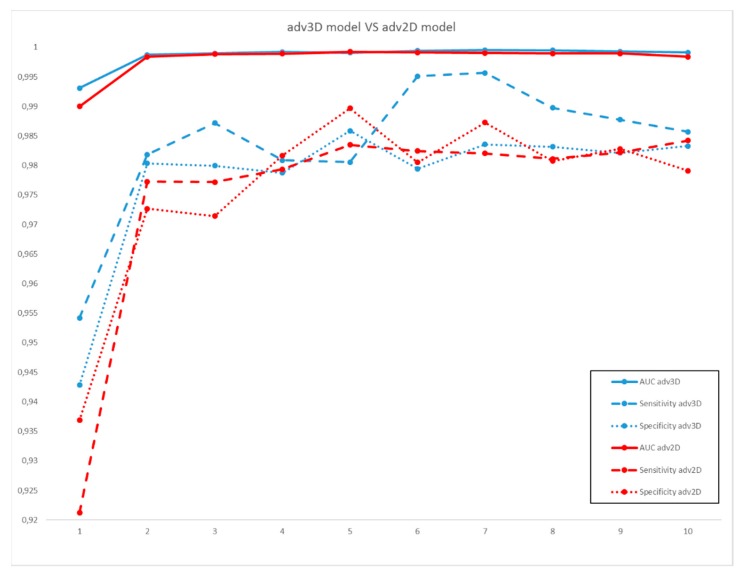
Area under the receiver operating characteristics curve (AUC) (full line), sensitivity (dashed line), and specificity (dotted line) of the multivariable models for adv3D (in blue) and adv2D (in red), for model orders from 1 to 10.

**Figure 2:**
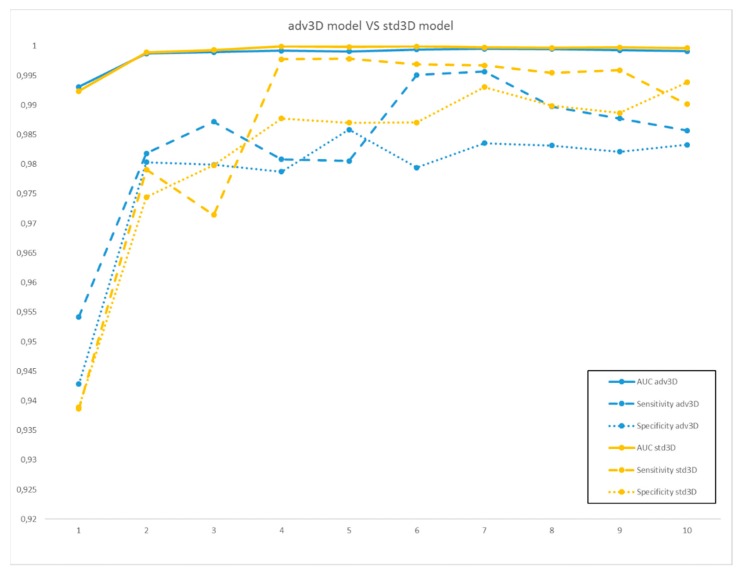
Area under the receiver operating characteristics curve (AUC) (full line), sensitivity (dashed line), and specificity (dotted line) of the multivariable models for adv3D (in blue) and std3D (in yellow), for model orders from 1 to 10.

For each reduced feature set, multivariable logistic regression models of order from 1 to 10 were obtained and their prediction performances for the different classification tasks are reported in Figure 1 and Figure 2, where the comparisons between adv3D/adv2D and adv3D/std3D are shown, respectively.

By inspecting the curves in Figure 1 and Figure 2, very similar and good results were obtained by the three classification models. In more detail, from the comparison between the adv3D and adv2D comparable results in terms of AUC, the sensitivity was higher for adv3D, except for model order 5; this was in contrast with specificity, which was higher on average for adv2D. On the other hand, the comparison between adv3D and std3D highlighted higher results for std3D, except for low order models.

For adv3D, the best model was order 7 based on the T2W mean, Max, Mad, Rms, ADC energy, Min, and K variance. Order 5 was chosen for adv2D based on T2W Mad, ADC mean, median, energy, and K^trans^ median. Finally, order 4 was selected for std3D based on T2W mean and Min, ADC energy, and Min.

## 3. Discussion

In this work, the added value of combining the radiomic processing approach with the advanced DKI mathematical model for PCa detection was investigated. The main aim was to compare a standard radiomic model built using T2W and ADC with an advanced one which included DKI and quantitative DCE pharmacokinetic parameters in terms of PCa diagnostic accuracy. In addition, we evaluated the differences found in prediction performances when using 2D ROIs or 3D VOIs.

The obtained results of AUC, sensitivity, and specificity were extremely high for all the classification tasks tested (PI-RADS 4–5 vs. HT on adv3D, PI-RADS 4–5 vs. HT on adv2D, and PI-RADS 4–5 vs. HT on std3D), reaching values up to 0.99 for AUC and 0.98 for both sensitivity and specificity. These performances were comparable to those obtained by Chen et al. [27] in their logistic regression model, which was built by incorporating T2W sequences and ADC maps to classify PCa vs. non-PCa tissues. However, in their work, Chen et al. included also shape features, which were deliberately not considered in our models in order to avoid possible biases in feature values introduced by the delineation of HT VOI/ROI which did not follow anatomical boundaries, as in the case of tumor lesions.

Looking at the variables included in the best predictive model chosen for each classification task, the recurrence of ADC energy as a common feature was observed. Interestingly, this index of tumor heterogeneity was previously found to be a promising quantitative imaging biomarker for characterizing cancer imaging phenotypes, since it was associated with tumor gene expression, tumor metabolism, tumor stage, patient prognosis, and treatment response in several studies on different cancer types [33].

To the best of our knowledge, only a few previous works [29,30,31] performed radiomic studies on prostate cancer, including DKI model parameters, to detect clinically relevant PCa and to evaluate PCa aggressiveness. Even though a direct comparison is not directly applicable, considering the different populations and imaging approaches, some parallels can be carefully drawn with the work by Wang et al. [29], which partially shared our aim and also considered the Toft perfusion model. Those authors found that the best ranked features for PCa detection were extracted from D and K, while pharmacokinetic features were not highly ranked in their classification model. Similarly, in our adv3D model, the majority of the best ranked features (see Table 1) were extracted from DKI and ADC (which was not included in the models by Wang et al. [29]), while few features were derived from the Toft parameters. 

In this study, looking in particular at the comparison between standard and advanced models, radiomic approaches based on a standard feature set (T2w and ADC) led to a predictive model with higher AUC, sensitivity, and specificity values than those of the predictive model based on the advanced feature set. It should be noted that the DKI and DCE features, even though they contributed to the features set after the first reduction step, survived only to a minor extent in the final selected models and minimally contributed K variance in the adv3D model and K^trans^ median in the adv2D model. These results suggest that the inclusion of radiomic features derived from DCE and DKI models does not provide a clear added value for PCa detection. On one hand, this would justify the choice to exclude DCE from dominant sequences for PI-RADSv2 score assessment, which is still a topic of discussion [34,35,36]; on the other hand, this would be food for thought regarding the complex debate of the financial benefits of including time- and computational-demanding DKI acquisition in prostate mpMRI acquisition protocol [14]. Regarding the comparison of the 3D and 2D advanced models, high diagnostic performances were shown by both advanced models, although the sensitivity was slightly lower and the specificity higher for the model built using 2D ROI, even though the resulting AUCs were similar and above 0.99, thereby suggesting great accuracy for the quantitative parameters extracted from VOI. This confirmed the hypothesis that tridimensional regions of interest allow for a more complete description of the lesion [37] and increased the number of points included in the statistical feature computation, thereby leading to results that were, in principle, more reliable and less vulnerable to sampling errors [38].

Our study presents several limitations. First of all, even if the lesions selected as PI-RADS 4–5 were confirmed during biopsy, uncertainty in histological radiological correlations may still remain when determining HT VOIs performed on the basis of radiological images. Such uncertainties could be ruled out using MR-guided biopsy techniques, which could adopt the advantages of new technologies, such as 3D printing, and could be used to develop personalized mold from diagnostic images to obtain histopathological slices exactly corresponding to the acquired slice in mpMRI, as an example [39]. In addition, due to the small sample size (< 100 cases), we could not evaluate the prediction accuracy for a nonbinary classification task (i.e., the additional inclusion of PI-RADS 3 lesions), neither could we test the reproducibility of the proposed method in a separate validation group of patients.

## 4. Materials and Methods 

### 4.1. Patient Cohort

The study was approved by the Institutional Review Board (9/19). All mpMRI data acquired from March 2017 to August 2018 from a single center were retrospectively checked. A total of 65 patients were selected according to the following inclusion criteria: PI-RADS 4–5 at mpMRI, PSA > 4 ng/mL, age older than 18 years at the time of the study, and available results of biopsy. Exclusion criteria included inadequate MR images and unbiopsied lesions. 

### 4.2. MR Imaging

mpMRI examinations were performed on a 3T Biograph mMR (Siemens Healthcare, Erlangen, Germany) with a body surface coil. The imaging protocols included a T2W (Repetition Time [TR] = 4010 ms, Echo Time [TE] = 112 ms, in plane field of view [FOV] = 200 × 200 mm^2^, number of slices = 26, resolution = 0.6 × 0.6 mm2, slice thickness = 3 mm), DWI (TR = 7000 ms, TE = 86 ms, in plane FOV = 260 × 220 mm^2^, number of slices = 26, resolution = 2 × 2 mm^2^, slice thickness = 3 mm, b values = 0, 250, 500 (4 averages), 1000, 1500 (6 averages), 2000, 2500 (8 averages) s/mm^2^), 6 gradient echo Volumetric Interpolated Breath-hold Examination (VIBE) sequences at variable flip angles (FAs) for T1 mapping (TR = 5.58 ms, TE = 1.83 ms, FAs = (2°, 5°, 8°, 12°, 15°, 20°), in plane FOV = 243 × 260 × 80 mm^3^, slice gap 20%, resolution = 1.4 ×1.4 × 3.0 mm^3^), and a dynamic scan with 60 consecutives phases with a VIBE sequence (TR = 5.58 ms, TE = 1.83 ms, FA = 20°, FOV = 243 × 260 × 80 mm^3^, resolution = 1.4 × 1.4 × 3.0 mm^3^, temporal resolution = 9 s/phase). Intravenous contrast injections started at the end of the first phase of dynamic scan at a dose of 0.1 mmol/kg of body weight and at the highest rate compatible with the patient’s age and compliance (up to 5 mL/s) 

### 4.3. PI-RADS

The PI-RADS assignment was performed by two radiologists experienced in urogenital imaging. They inspected, in consensus, T2W, DWI, ADC maps, and DCE images in order to identify prostatic lesions in accordance to PI-RADS v2 guidelines [40]. Of the PI-RADS 4 and PI-RADS 5 lesions, 100% were confirmed to be cancer on biopsy. 

### 4.4. ADC and DKI Maps Calculation

ADC maps were computed using the in-line software of the Biograph scanner, selecting b-values from 0 to 1500. DKI maps, i.e., D and K, were computed using a voxel-wise fitting procedure implemented in Matlab (The MathWorks Inc., Natick, MA, USA). 

### 4.5. Pharmacokinetic Map Calculation

Pharmacokinetic maps were obtained with the commercial software Tissue 4D (Siemens Healthcare, Erlangen, Germany). After an automated step of motion correction of the VIBE sequences at variable FAs with the dynamic VIBE sequence, the Toft model [41] was chosen for pharmacokinetic parameter calculations. The arterial input function (AIF) used for the analysis was set to “intermediate”, on the basis of population-based AIFs built in Tissue 4D. Finally, 3D maps of K^trans^, K_ep_, v_e_, and iAUC were obtained [23]. 

### 4.6. Image Preprocessing

Before feature extraction, some preprocessing steps were performed for each subject, i.e., the image acquired at b = 0 was non-rigidly coregistered to the T2W image in order to correct for spatial distortion typical of DWI acquisition. The registration was performed using the Elastix software (v. 4.9.0 [42]), and the resulting transform was used to warp the volumes acquired at the other b-values and the corresponding ADC, D, and K maps. In addition, K^trans^, K_ep_, v_e_, and iAUC maps were resampled to match the resolution of the T2W image.

### 4.7. VOI/ROI Segmentation

3D VOI segmentations were manually obtained. Two experienced radiologists were asked to consensually draw the 3D VOI in the biopsied lesions with PI-RADS 4–5 and in HT on T2W images, while also looking at the b = 1000 coregistered volume. During the segmentation procedure, the radiologists were blinded to both the histological results and all clinical information relative to the retrospective prostate MR images. The segmentation was done using in-house developed software for region labeling. The 2D ROIs were automatically obtained in Matlab from 3D VOIs, with the 2D section with the longest major axis chosen for each. 

### 4.8. Feature Extraction

The first order features were extracted from T2W, ADC, D, K, K^trans^, K_ep_, v_e_, and iAUC for the 3D VOIs and 2D ROIs. First, each image was normalized, limiting its dynamics within the segmentation to μ ± 3σ [43], then 13 first order features were extracted from the intensity histogram computed on 256 bins, namely, energy, entropy, kurtosis, maximum (Max), mean, mean absolute deviation (Mad), median, minimum (Min), root mean square (Rms), skewness, standard deviation (Std), uniformity, and variance.

In addition, second order features were also computed for the T2W and ADC images. Gray Level Co-occurrence Matrix (GLCM) [44], which was computed by 3D analysis of the tumor region with 26-voxel connectivity, was chosen, simultaneously taking into account the neighboring properties of the voxels in all 3D directions [45] after image quantization on 32 grey levels. The obtained features included energy, contrast, entropy, homogeneity, correlation, sum average, variance, dissimilarity, and auto correlation.

### 4.9. Multivariable Analysis

Three classification tasks were analyzed: PI-RADS 4–5 vs. HT on 3D VOI in the adv3D, which was computed using first order features from T2W, ADC, D, K, K^trans^, K_ep_, v_e_, and iAUC, PI-RADS 4–5 vs. HT on 2D ROI in the adv2D, and PI-RADS 4–5 vs. HT on 3D VOI in the std3D, which was computed using the first and second order features from T2W and ADC. 

The multivariable predictive models were obtained following the method described by Vallières et al. [45], using an imbalance-adjusted bootstrap resampling (IABR) on 1000 samples at each step.

First, from the large initial set of features, a reduced feature set of 25 features was computed through a stepwise forward feature selection scheme for each classification task. The first feature was chosen as the one that maximized Spearman’s rank correlation regarding the outcome. Then, the features were added one at a time to maximize a linear combination of Spearman’s rank correlation (between the feature and the outcome) and the Maximal Information Coefficient (between the feature and the features that were yet to be included in the reduced set) [46]. 

Then, from the reduced feature set, logistic regression models of order i from 1 to 10 that would best predict the outcome under investigation were obtained using another stepwise forward feature selection. This procedure involved adding features that maximized the 0.632 + bootstrap area under the receiver operating characteristic curve (AUC) [47] to the ith model one by one. 

Finally, for each classification task, the prediction model was obtained by choosing the order that maximized the AUC and computing the final model logistic regression coefficients for the aforementioned combination of features using IABR.

## 5. Conclusions

In this work, we proposed a radiomic approach to differentiate between PI-RADS 4–5 lesions and HT using different prediction models. The proposed comparisons, including one between models constructed from standard mpMR acquisition protocol (including ADC and T2W) and advanced acquisition protocol (with the addition of DCE and DKI) and another between models derived from features extracted from 3D VOIs or 2D ROIs, provided interesting insights into these sensitive discussion topics amongst the scientific community. This work paved the way toward further studies of these topics, potentially evaluating nonbinary outcomes, tumor aggressiveness, and reproducibility of the computed features in wider cohorts of patients. More extensive work could enable the scientific community to more confidentially suggest guidelines regarding the choice of the most confident, but not redundant, acquisition protocol, and of the most appropriate postprocessing pipeline to accurately detect and characterize PCa.

## Figures and Tables

**Table 1:** Reduced feature set for each classification task. For each feature, the image from which it was extracted is indicated. For std3D, whether it is a first or second order feature and the feature name are also indicated. Abbreviations: T2W = T2-weighted; ADC = Apparent Diffusion Coefficient; D = diffusion coefficient of Diffusion Kurtosis Imaging (DKI) model; K = deviation from normal distribution coefficient; iAUC = initial area under the enhancement curve; v_e_ = volume of extravascular extracellular space per unit volume of tissue; K_ep_ = ; K^trans^ = ; Max = maximum; Mad = mean absolute deviation; Min = minimum; Rms = root mean square; Std = standard deviation; GLCM = Gray Level Co-occurrence Matrix.

adv3D	adv2D	std3D
D—Mean	ADC—Rms	ADC—Mean
D—Energy	T2W—Energy	ADC—Energy
iAUC—Median	K^trans^—Median	ADC—GLCM Auto Correlation
v_e_—Min	T2—Std	ADC—Max
T2W—Max	K—Mad	ADC—Min
K—Mad	K—Std	T2W—Max
ADC—Max	D—Max	T2W—Std
ADC—Min	T2W—Max	T2W—Mean
T2W—Std	ADC—Energy	ADC—Rms
K—Std	D—Mean	ADC—Median
ADC—Energy	ADC—Max	T2W—Variance
K—Variance	D—Energy	T2W—Energy
T2W—Variance	T2W—Variance	T2W—Rms
T2W—Rms	K—Variance	ADC—GLCM Sum Average
D—Max	D—Rms	T2W—Median
D—Min	D—Median	T2W—Mad
T2—Mad	ADC—Mean	T2W—GLCM Correlation
K_ep_—Median	T2W—Mean	ADC—Skewness
T2W—Energy	K—Rms	T2W—GLCM Homogeneity
T2W—Mean	ADC—Median	T2W—Uniformity
D—Rms	T2W—Mad	T2—Entropy
K^trans^—Min	K^trans^—Mean	T2—GLCM Dissimilarity
K^trans^—Mean	T2W—Median	T2—Min
D—Median	iAUC—Median	ADC—Uniformity
ADC—Mean	T2W—Rms	ADC—GLCM Correlation
